# ClimateQUAL: Advancing Organization Health, Leadership, and Diversity in the Service of Libraries

**DOI:** 10.5195/jmla.2019.698

**Published:** 2019-07-01

**Authors:** Elizabeth Connor

**Affiliations:** connore1@citadel.edu, Daniel Library, The Citadel, Charleston, SC

Assessment is an essential element of modern library operations. Librarians are accustomed to measuring units of service and using those data points to demonstrate value, return on investment, and impact on institutional outcomes. Charles B. Lowry is the former executive director of the Association of Research Libraries (ARL) and former dean of libraries at the University of Maryland at College Park. Lowry has assembled an impressive group of contributors to explain the value of a specific tool, ClimateQUAL.

Readers may be familiar with the utility of LibQUAL+ for benchmarking and addressing patron perceptions of library service quality. ClimateQUAL was based on the University of Maryland’s groundbreaking Organizational Climate and Diversity Assessment (OCDA).

ClimateQUAL is useful for gauging workplace climate including organizational health, diversity, and inclusion. Librarians who are committed to organizational development and continuous improvement will appreciate ClimateQUAL as an important management tool. Librarians who are focused on meaningful assessments will want to measure patron perceptions and library culture. In a culture of assessment, it makes sense to use LibQUAL+ and ClimateQUAL to measure both.

ClimateQUAL’s underlying premise is that healthy organizations are more likely to provide excellent services. Organizational strife can be detrimental to patron perceptions of service quality. Organizational diversity can improve organizational operations. As with LibQUAL+, ClimateQUAL survey results can be used to improve a library’s climate and customer services, if the organization is committed to making changes based on findings.

In a nutshell, ClimateQUAL can help gauge how well a library communicates expectations and rewards related to fairness, innovation, customer service, demographic diversity, and teamwork by surveying staff teams. Chapter 1 provides an overview of OCDA and the eventual development of ClimateQUAL. Chapter 2 describes the theory of healthy work attitudes and workplace climate. Chapter 3 discusses the centrality of library leadership, particularly “Authentic Leadership and Leader-Member Exchange” (LMX) on healthy workplaces. Chapter 4 explains the relationship of climate (as measured by ClimateQUAL) on service quality (as measured by LibQUAL+). Chapter 5 details how libraries can interpret and use the survey results to make improvements. Chapter 6 outlines the improvement strategies used by seven libraries that have administered ClimateQUAL more than once. Chapter 7 delves into the relationship between diversity and library effectiveness. Chapter 8 provides the perspective of ClimateQUAL as administered in four academic libraries in the United Kingdom, showing that this tool is applicable to other cultural settings. The work includes a useful index and contributor biographies.

Librarians who focus on continuous improvement and care to learn more about how workplace climate influences service quality will appreciate this work. It is highly recommended.

